# Is Perceived Exertion a Useful Indicator of the Metabolic and Cardiovascular Responses to a Metabolic Conditioning Session of Functional Fitness?

**DOI:** 10.3390/sports7070161

**Published:** 2019-07-04

**Authors:** Ramires Alsamir Tibana, Nuno Manuel Frade de Sousa, Jonato Prestes, Dahan da Cunha Nascimento, Carlos Ernesto, Joao Henrique Falk Neto, Michael D. Kennedy, Fabrício Azevedo Voltarelli

**Affiliations:** 1 Graduate Program in Health Sciences, Faculty of Medicine, Federal University of Mato Grosso (UFTM), Cuiabá 78000, MT, Brazil; 2 Laboratory of Exercise Physiology, Faculty Estacio of Vitoria, Vitoria 29010, ES, Brazil; 3 Graduation Program on Physical Education, Catholic University of Brasilia, Brasilia 04534, DF, Brazil; 4 Athlete Health Lab, Van Vliet Complex, Faculty of Kinesiology, Sport, and Recreation, University of Alberta, Edmonton, AB T6G 2H9, Canada

**Keywords:** CrossFit, high-intensity functional training, extreme conditioning programs, overtraining, overreaching

## Abstract

The purpose of this study was to assess whether the self-regulation of training intensity based on rating of perceived exertion (RPE) is a reliable method to control the intensity during metabolic conditioning sessions of functional fitness. In addition, the relationship between RPE and the changes in heart rate, number of repetitions, and lactate responses was also analyzed. Eight male participants (age 28.1 ± 5.4 years; body mass 77.2 ± 4.4 kg; VO_2_ max: 52.6 ± 4.6 mL·(kg·min)^−1^ completed two sessions (five to seven days apart), in a randomized order, under different conditions, as follows: (1) all-out (ALL), or (2) self-regulation of intensity based on an RPE of six (hard) on the Borg CR-10 scale (RPE6). The rating of perceived exertion, lactate (LAC), and heart rate (HR) response were measured before, during, and immediately after the sessions. The RPE and LAC during the all-out sessions were higher (*p* < 0.0005) than the RPE6 session for all of the analyzed time points during the session. There was no difference in the HR area under the curve for the all-out and RPE6 sessions. The average number of repetitions performed was lower (*p* ≤ 0.009) for the RPE6 session (190.5 ± 12.5 repetitions) when compared to the all-out session (214.4 ± 18.6 repetitions). There was a significant correlation between the RPE and LAC (*p* = 0.005; r = 0.66; large) and number of repetitions during the session (*p* = 0.026; r = 0.55; large). No correlation was observed between the RPE and HR (*p* = 0.147; r = 0.380). These results indicate that the self-regulation of intensity of effort based on the RPE may be a useful tool to control the exercise intensity during a metabolic conditioning session of functional fitness.

## 1. Introduction

Functional-fitness training (FFT), also known as CrossFit, high-intensity functional training (HIFT), or extreme conditioning programs (ECP), is an exercise modality that contemplates a variety of training methods. Sessions are often classified as weightlifting (W), metabolic (M), or gymnastics (G), and utilize weightlifting/powerlifting exercises (e.g., clean and jerk, snatch, squat, deadlift, push press, bench press, and power clean), calisthenic bodyweight exercises (e.g., pull-ups, dips, push-ups, handstands, presses to handstands, pirouettes, kips, cartwheels, and muscle-ups), cardiovascular exercises (e.g., rowing, biking, and running), sprints, and flexibility exercises, depending on the goal of the session and the fitness components that are to be targeted [1,2]. FFT programs are usually planned so that a combination of each type of session is performed on a weekly basis, simultaneously enhancing multiple fitness components, such as aerobic power and anaerobic capacity, muscular endurance, strength, and power [2]. Participants commonly perform three to five whole-body exercise sessions a week, with most, if not all, involving some form of conditioning [3]. The metabolic training sessions are often performed either as a single mode of exercise focusing on a cardiovascular exercise, or utilizing a combination of exercise methods in order to maximize physiological stress and the purported training adaptations [4]. While not all sessions are performed in an all-out manner, this type of effort is common in FFT (from the most famous FFT program), as the sessions are frequently performed as rounds for time (RFT) or as many rounds as possible (AMRAP), encouraging participants to complete the highest amount of work possible in a set period of time [3,5].

Previous research has shown that the metabolic conditioning sessions of functional-fitness training resulted in increased acute oxidative stress [6]; high metabolic, inflammatory [7], and cardiovascular responses; elevated perceived exertion [8]; and increased sympathetic nervous system markers (i.e., plasma epinephrine and norepinephrine) [9]. As a result of the increases in oxidative and inflammatory markers [6,7], and the extreme effort associated with FFT, some studies have raised concerns about a tendency for the development of symptoms of overtraining in functional fitness practitioners [10,11]. For example, Drake, Smeed, Carper and Crawford [10] found that four weeks of FFT led to a state of functional overreaching in some participants, and that non-functional overreaching could be developed if the high intensity associated with training was maintained after the four weeks of study. Similarly, Drum, Bellovary, Jensen, Moore and Donath [11] demonstrated a high presence of severe post-exercise symptoms during a CrossFit program, such as excessive fatigue, muscle soreness, muscle swelling, and limited muscle movement during workouts as a result of the extreme intensity of the session. Despite the evidence that finds extreme metabolic conditioning sessions can lead to severe post-exercise symptoms of fatigue, the current literature regarding the methods of monitoring and controlling training intensity during these sessions is limited.

In this context, a correct control and prescription of the training intensity can minimize the deleterious effects that have been shown to occur following metabolic sessions or periods of intense training. Considering the wide variety of exercises used during such sessions (strength/power, gymnastics, and endurance), controlling training intensity is a challenge. The Borg CR-10 scale, called the rate of perceived exertion (RPE) scale [12], has been widely used to determine the intensity during different modalities of exercise, including resistance training [13], high-intensity interval exercise [14], and swimming [15]. The use of RPE has been shown to be related to physiological markers, such as maximal oxygen consumption (VO_2_ max), lactate, and ventilatory thresholds, and can be used as a surrogate for heart rate to understand the heart rate response to a specific exercise intensity. However, the validity and utility of RPE for prescribing and self-regulating training intensity during metabolic conditioning sessions of FFT has not been studied. Furthermore, the relationships between the metabolic and cardiovascular responses and RPE during these sessions in FFT have also not been established.

Thus, the aim of the present study was to examine whether RPE could be used as a method to prescribe exercise intensity during metabolic conditioning sessions in FFT. Secondly, a comparison between the physiological responses of the RPE-prescribed session to that of the typical all out conditioning session was performed to assess the difference in the total work performed. It was hypothesized that participants would be able to self-regulate intensity when a target RPE was prescribed, and that the metabolic and cardiovascular response, in addition to the total work done, would be lower when the intensity is regulated via RPE.

## 2. Materials and Methods

### 2.1. Participants

Eight male members of the functional fitness community (age 28.1 ± 5.4 years; body mass 77.2 ± 4.4 kg; VO_2_ max: 52.6 ± 4.6 mL·kg·min)^−1^; 3.8 ± 1.4 years of experience) were recruited through advertisements. All of the subjects were free of injury and known illness, were not using drugs to enhance performance, and had a minimum of six months of FFT experience. The subjects were advised to sleep between six and eight hours the night before each experimental session; to maintain their regular hydration and food consumption habits; to avoid any exercise in the 48 h before the experimental sessions; and to avoid smoking, alcohol, and caffeine consumption 24 h before the experimental session. All of the subjects signed an informed consent document, and the study was approved by the University Research Ethics Committee for Human Use (2.698.225/Universidade Estácio de Sá/UNESA/RJ) and conformed to the Helsinki Declaration on the use of human participants for research.

### 2.2. Experimental Design

**Figure 1:**
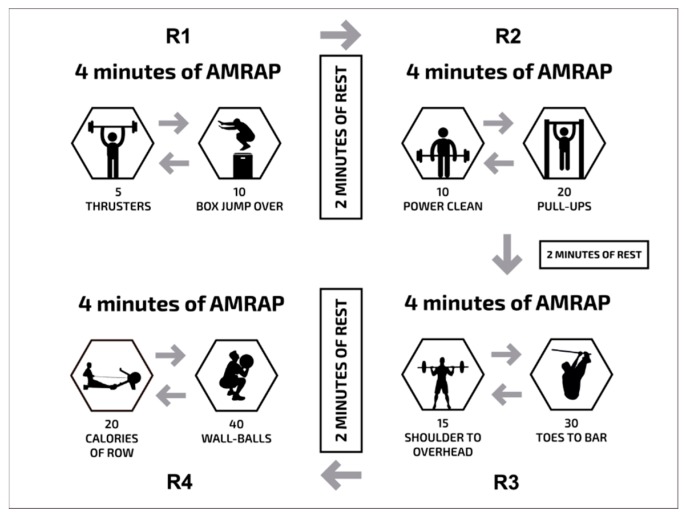
Metabolic conditioning: 4 min of as many rounds as possible (AMRAP) of five thrusters, and 10 box jump over (round 1); 2 min of rest; 4 min of AMRAP of 10 power clean, and 20 pull-ups (round 2); 2 min of rest; 4 min of AMRAP of 15 shoulder to overhead, and 30 toes to bar (round 3); 2 min of rest; and 4 min of AMRAP of 20 calories of row, and 40 wall ball (round 4).

**Figure 2:**
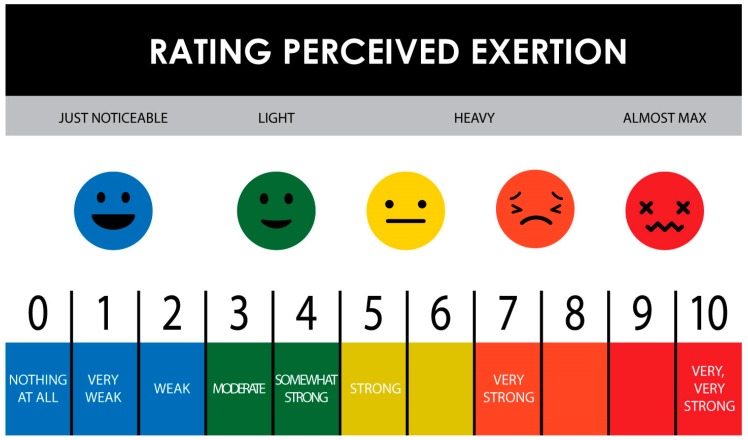
Rate of perceived exertion table made available to participants during the metabolic session of functional fitness training. Adapted from the literature [12,18,19].

The subjects completed a metabolic conditioning session (five to seven days apart) in a randomized fashion under two different conditions, namely: (1) all-out (ALL) or (2) the self-regulation of intensity based on an RPE of six (hard) on the Borg CR-10 scale (RPE6). The all-out and RPE-based autoregulation sessions were as follows: 4 min of as many rounds as possible (AMRAP) of five thrusters (60 kg), and 10 box jumps over (round 1); 2 min of rest; 4 min of AMRAP of 10 power clean (60 kg), and 20 pull-ups (round 2); 2 min of rest; 4 min of AMRAP of 15 shoulder to overhead (60 kg), and 30 toes to bar (round 3); 2 min of rest; and 4 min of AMRAP of 20 calories of row, and 40 wall ball (9 kg; round 4; Figure 1). During the all-out workout, the subjects were instructed to complete the maximum number of repetitions possible for each round. The RPE-based autoregulation session consisted of performing the same activity, but with the participants told to self-regulate the intensity of their session based on a perception of effort of six (hard) on the Borg CR-10 scale. During the session, the subjects were instructed to take more breaks if needed, or to just “slow down” the execution of their exercises to keep the perception of effort at six (hard). No changes to the weights were performed during the sessions. The Borg CR-10 scale was printed and available to the participants as a visual reminder of the prescribed target intensity (Figure 2).

### 2.3. Blood Lactate

Capillary blood samples were collected through a transcutaneous puncture on the medial side of the tip of the middle finger using a disposable hypodermic lancet. The blood lactate (LAC) concentrations were measured before and immediately after 4, 10, 16, and 22 min in each protocol of exercise. The LAC was determined by photometric reflectance on a validated Portable Accutrend Plus system (Roche, Sao Paulo, Brazil).

### 2.4. Heart Rate (HR)

Continuous monitoring of HR during the experimental sessions was done with the use of a Polar H10 HR-monitor (Polar Electro Oy, Kemple, Finland), with a recording interval of 1 s. The maximal heart rate was obtained from the 2-km row test that was used for the indirect assessment of the maximal oxygen uptake [16] of the subjects. The 2 km row test consisted in rowing 2 km with the maximal effort (power) possible. During the test, continuous monitoring of the HR was done, and the maximum HR during the test was used as the maximum HR of the subject.

### 2.5. Rating of Perceived Exertion (RPE)

The data were collected as previously described by Tibana, et al. [17]. The RPE was measured before, during (immediately after 4, 10, and 16 min), and immediately after exercise, by the RPE CR10 Borg scale adapted from Foster, et al. [18] and Morishita, et al. [19], an instrument composed by a Likert type scale of 11 points, varying from 0 to 10, initiated with “very, very light“ and terminated with “very, very hard “. The following instructions were used to ensure that each participant clearly understood what the RPE scale was and how it was to be used to regulate their exercise intensity. First, the RPE was explained to the subjects individually, according to the recommendations from Foster et al. [18]. Secondly, the following information was verbally provided: “The perceived exertion is defined as the effort intensity, stress, discomfort, and fatigue felt during exercise. Utilize the numbers of this scale to report how your body feels during exercise. The number zero in the scale describes “minimal effort” and represents your lowest imaginable effort. The number 10 describes “maximum effort” and represents the highest imaginable effort. If you feel an exertion between extremely easy and maximum effort indicate a number between 0 and 10. There are no right or wrong numbers. The verbal descriptors may help you to choose a number” [18].

During the sessions, a printed version of the RPE scale (enlarged poster size-dimensions) was fixed on a wall with tape so that the subjects could visualize the scale at all times. During the anchoring procedure, the subjects were instructed by another evaluator that was present in the testing room to describe their effort using the RPE scale. The subjects also received a copy of the scale with the respective instructions for anchorage. This was provided for subjects to read during the general warm-up for each session [18].

### 2.6. Statistical Analysis

Data are expressed as mean ± standard deviation (SD). The data distribution was assessed with the Shapiro–Wilk test, and all of the variables presented normal distribution. A two-way repeated measures ANOVA (sessions x time) was used to compare the LAC, HR, and RPE between the RPE6 and all-out sessions. Mauchly’s test was used to assess the assumption of sphericity, and when not met, a Greenhouse-Geiser adjustment was used to determine the significance of the ANOVA tests. Tukey’s post-hoc test with a Bonferroni correction was applied in the event of significance. A paired sample t-test was used to compare the number of repetitions and the area under the curve of LAC, HR, and RPE generated during the 22 min of the RPE6 and all-out sessions. The Spearman product moment correlation was used to evaluate the relationship between RPE and LAC, and RPE and HR (because the AUC of RPE did not present as a normal distribution). Instead of a fixed time point of the study variables, the area under the curve for all of the correlations (RPE, LAC, and HR) during the all-out and RPE6 sessions was utilized. The magnitude of the correlations was classified as follows: r ≤ 0.1 trivial; 0.1 < r ≤ 0.3 small; 0.3 < r ≤ 0.5 moderate; 0.5 < r ≤ 0.7 large; 0.7 < r ≤ 0.9 very large; r > 0.9 almost perfect [20]. The achieved power of the sample size was calculated based on the interaction of the RPE between the all-out and RPE6 sessions. The effect size (f) was 0.312 and the achieved power was 0.810. The level of significance was *p* ≤ 0.05, and all of the analyses were performed using SPSS version 20.0 (Somers, NY, USA).

## 3. Results

### 3.1. Number of Repetitions Performed

The average number of repetitions was significantly lower for the RPE6 session (190.5 ± 12.5 repetitions) than for the all-out session (214.4 ± 18.6 repetitions). However, as shown in Table 1, the differences in work completed in each round varied. Specifically, as shown in Table 1, more reps were completed in the all-out condition for R1 and R2. In R3, the RPE6 condition completed more reps when compared with the all-out condition, and in R4, the average difference between the two sessions was fewer than two reps. Table 1 presents the results of each round of FFT sessions, as well as the percentage change in work done between the rounds. Lastly, only one participant completed more reps in the RPE6 condition than the all-out session.

### 3.2. Rating of Perceived Exertion

**Figure 3:**
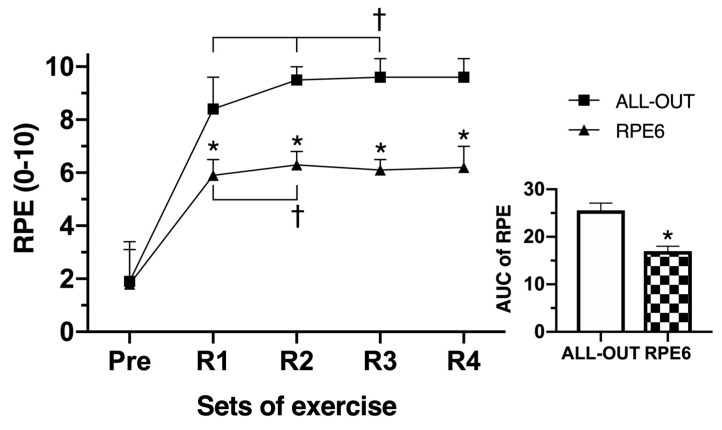
Ratings of perceived exertion (RPE) before and at the end of round 1 (R1), R2, R3, and R4, and the area under the curve (AUC) of the RPE during the functional fitness sessions. Differences between sessions: * *p* ≤ 0.05 for the all-out; differences between time: † *p* ≤ 0.05 for R1 and R2 in all-out and R1 in RPE6.

A significant two-way interaction between functional fitness sessions and time was found for RPE (*p* < 0.0005; Figure 3). The RPE during the all-out session was significantly higher than the RPE6 session at each time point. There was a significant increase in RPE from rest to R1, R2, and R3 in the all-out condition, and from rest to R1 and R2 during the RPE6 session (Figure 2). However, the global RPE, as determined via the 22 min area under the curve for RPE during all-out, was significantly higher (*p* < 0.0005) than during the RPE6 session.

### 3.3. Blood Lactate Concentration

**Figure 4:**
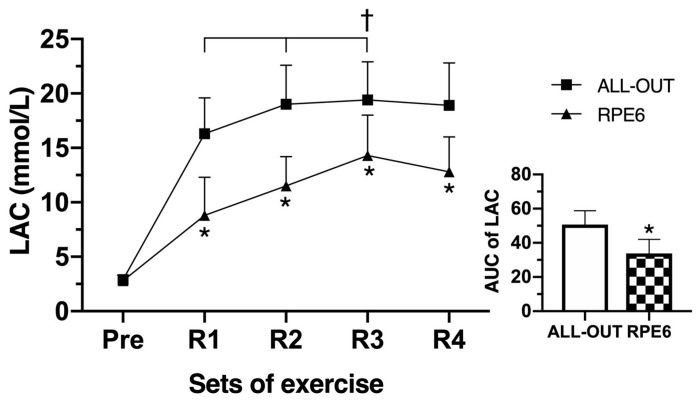
Blood lactate concentration (LAC) before and at the end of round 1 (R1), R2, R3, and R4, and area under the curve (AUC) of the LAC during the functional fitness sessions. Differences between sessions: * *p* ≤ 0.05 for all-out; differences between time: † *p* ≤ 0.05 for before, R1 and R2 in both all-out and RPE6.

There was a statistically significant two-way interaction between the session and time for LAC (*p* < 0.0005; Figure 4). The LAC during the all-out session was significantly higher than the RPE6 session at each time point (R1, R2, R3, and R4) The LAC increased until R3 during the all-out and RPE6 sessions, where R1 was different than rest, R2 was greater than R1, and R3 was greater than R2 for both all-out and RPE6. The LAC area under the curve during the all-out session was significantly higher (*p* = 0.002) than during RPE6.

### 3.4. Heart Rate

**Figure 5:**
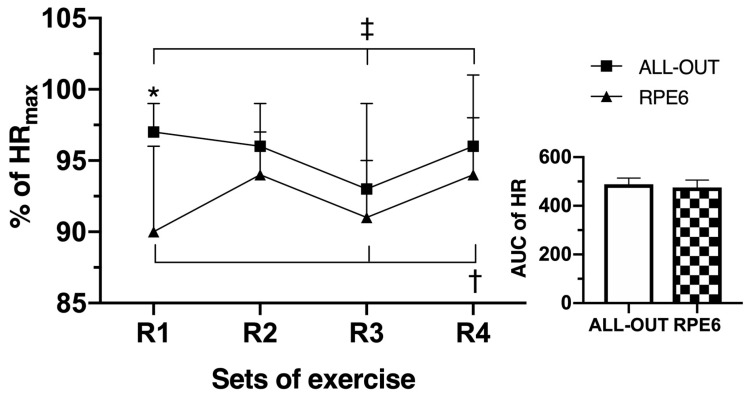
Percentage of maximal heart rate (HRmax) at the end of round 1 (R1), R2, R3, and R4, and the area under the curve (AUC) of the HRmax during the functional fitness sessions. Differences between sessions: * *p* ≤ 0.05 for RPE6; differences between time: † *p* ≤ 0.05 for R1 and R3 in RPE6; ‡ *p* ≤ 0.05 for R1 and R4 in all-out.

Figure 5 shows the % of maximal heart rate (HRmax) during the functional fitness sessions. There was a significant interaction between the functional fitness sessions and time for the % of HRmax (*p* = 0.024). The % of HRmax during the all-out session was significantly higher (*p* = 0.005) than the RPE6 session only at the end of R1. No statistically significantly differences (*p* = 0.156) for the AUC were observed between thw all-out and RPE6 sessions (Figure 4). For the RPE6 session, the % of HRmax at R4 was greater than R1 (*p* = 0.016) and R3 (*p* = 0.030). During the all-out, the % of HRmax was the greatest at R1 (*p* = 0.036) and R4 (*p* = 0.043) compared with R3.

### 3.5. Correlations between RPE and Physiological Variables

A statistically significant correlation was observed between the RPE and LAC (*p* = 0.005; r = 0.665; large), and between the RPE and the number of repetitions performed during the session (*p* = 0.026; r = 0.555; large). No correlation was observed between the RPE and HR (*p* = 0.147; r = 0.380).

## 4. Discussion

The results presented in this study support the hypothesis that RPE could be used to regulate the intensity of metabolic conditioning sessions in trained men. Moreover, the results demonstrated the following: (1) the RPE and LAC during the all-out session were higher than the RPE6 at all of the time points; (2) the all-out condition leads to too much undue fatigue in latter portion of the session, as seen by a dramatic drop in the number of repetitions performed; (3) the % of HRmax during the all-out session was significantly higher than the RPE6 session only at the end of R1, and similar during all-out and RPE6 conditions when analyzed for the AUC; and that (4) the RPE is significantly correlated to the LAC and the number of repetitions performed.

Functional-fitness has been increasingly growing in popularity, as it is considered a more enjoyable form of exercise when compared with traditional aerobic and resistance training [21]. In addition, it is done in a shorter period of time, while inducing similar positive outcomes in strength [22], performance [23], and body composition [24] compared with longer duration, traditional resistance, and aerobic type sessions. To the best of our knowledge, this is the first study designed to examine whether RPE is a viable tool for controlling the intensity of a metabolic training session in trained men. The findings in this study corroborate what has been reported in other investigations that showed the viability of this method in several exercise methods and sporting disciplines, including resistance training [13], high-intensity interval training [14], and swimming [15]. For example, Ciolac et al. [14] found that HR response and walking/running speed were not different between high-intensity interval training sessions prescribed and regulated by HR or RPE in young individuals. Similarly, Ceci and Hassmen [25] analyzed two testing sessions consisting of both treadmill and track exercise at three different intensities—at an RPE of 11 (light exertion), followed by a RPE of 13 (somewhat hard) and by an RPE of 15 (hard). The authors showed significant different values of HR, blood lactate, and velocity at the three RPE intensities, and concluded that RPE was an effective tool to regulate exercise intensity in physically active males. Therefore, while there is support in the literature that RPE is a useful tool to monitor and control the intensity of single mode, traditional, and high intensity interval training (HIIT) aerobic sessions, this study is the first to demonstrate that RPE can be effective in regulating the intensity of FFT sessions.

In addition, this study provides new insight into the perceptual and physiological responses of an all-out exercise bout in FFT. First, it was assumed that RPE would be maximum (RPE of 10) throughout this training session. However, it only rose to 10 in all the participants by round 3, indicating that even in all-out exercises, some regulation still occurs. While the difference between groups in %HR (R1) and LAC, and RPE for every round confirms the higher intensity of the all-out condition from the beginning of the session, it is possible that a longer training duration is needed for the RPE to reach maximum levels. A recent study provides support for this notion, as it has been shown that the perceived effort of the session increases with its duration [26]. Furthermore, while much debate still exists on the mechanisms that regulate physical activity, the notion that the brain is involved in pacing all forms of voluntary physical activity has been discussed in the literature [27]. Further investigations are required in order to assess if and how FFT participants pace themselves during all-out sessions.

The very large LAC response is understandable given the characteristic of the session and presents values that are very similar to other all-out style assessments, such as a 90 s Wingate, or all-out events, like flat-water kayaking races or track cycling. Furthermore, an all-out strategy leads to a greater number of repetitions performed overall when compared with a sub-maximal intensity prescription of “hard to very hard” (RPE6 session). However, if the session had gone longer, it is likely that the RPE6 session may have resulted in greater total reps completed compared with the all-out condition. As shown in Table 1, by R3, the RPE6 condition was completing more repetitions (41.9 ± 6.6 vs. 48.0 ± 1.9), with this trend likely continuing to additional rounds of work because of less accumulated fatigue in the early part of the workout compared with an all-out strategy.

Nevertheless, most of the metabolic conditioning sessions that are utilized in FFT are 8 to 20 min in duration [2]. Considering the findings of this study, it is possible that in sessions with these durations, an all-out strategy would lead to a greater amount of total work completed. As an increase in work capacity is one of the key benefits that have been advocated to occur with FFT [3], based on these results, it is possible that all-out sessions would lead to greater improvements in this characteristic. Given the higher workload and physiological stress experienced during the all-out session, other fitness components that have been advocated to improve with FFT, such as muscular endurance, aerobic power, and anaerobic capacity, would likely increase to a greater extent as well.

Still, regardless of the training method, the correct application of the training stimulus is one of the fundamental factors for positive physiological adaptations to occur, leading to a concomitant improvement in performance [28,29]. Excessive training performed at a high intensity will result in negative adaptations, including non-functional overreaching and/or overtraining. As previously mentioned, most FFT participants train three to five times per week, with most sessions being either dedicated to metabolic conditioning or having some component of it. In this context, it is possible that the frequent performance of all-out bouts leads to undue fatigue that can have negative consequences on participants’ health and performance. Indeed, it has been shown that functional fitness practitioners have a tendency to develop symptoms of overtraining [10,11]. This tendency can be explained by the fact that a single session of metabolic conditioning leads to increased acute oxidative stress [6], metabolic and inflammatory stress [7], high cardiovascular and RPE responses [8], and elevated sympathetic nervous system markers (i.e., plasma epinephrine and norepinephrine) [9]. When investigating high-intensity interval training, Seiler, et al. [30] demonstrated that training at higher intensities led to higher levels of autonomic nervous system fatigue, with recreational athletes requiring as much as 72 h to recover from these sessions. The authors suggested that two to three high intensity training sessions might be the limit of what can be performed on a weekly basis, in order to allow for proper recovery between sessions. In this context, it is possible that a similar limit might exist for FFT sessions. Moreover, Navalta, et al. [31] showed that repeated intense interval exercise (three consecutive days of an intermittent run protocol to exhaustion) elicited significant CD4+, CD8+, and CD19+ lymphocyte cell death and migration after the third day of running. Considering this, it would be recommended to incorporate a rest day or training with lower intensity (through RPE) to minimize immune cell modulations and reduce potential susceptibility to infections.

Considering the high level of physiological and perceptual stress reported in these sessions, and how the performance of frequent all-out metabolic conditioning sessions has the potential to lead to non-functional overreaching, perhaps the greatest finding in this study is that the RPE6 can be used to achieve a high workload with lower physiological stress, and thus, less undue fatigue when compared with an all-out session. This has important practical applications for novice and experienced FFT participants. Tibana, et al. [32] recently described the training preparation of an FFT athlete throughout 38 weeks of training and reported that the acute to chronic workload ratio of the athlete often fell outside what is considered the “safe zone”. By controlling the intensity of some of the training sessions, it is possible that training loads could have been managed so as to avoid moments with an improper progression in load between weeks.

In addition, while it has been shown that FFT does not pose a greater risk of injury when compared to traditional resistance training or other common types of physical activity [1], other health concerns for participants have been raised. Particularly, reports of exertional rhabdomyolysis, a condition characterized by muscle necrosis followed by the release of intracellular muscle contents into the circulation, have increased following FFT sessions [5,33]. As the condition occurs in response to non-familiar and/or excessive, prolonged, or repetitive exercise, coaches and practitioners can match their training prescription to the desired exertion level, minimizing the risk of such a negative outcome. Thus, the assertion that RPE is a reliable tool to control the intensity of FFT sessions ensures the safety and optimal progression of the participants’ programs, while helping to dispel some of the health concerns associated with FFT.

Lastly, despite its widespread use in other modes of exercise, it seems that FFT should not be prescribed based on a percentage of maximal heart rate, as there was no difference in HR between groups (AUC). Although HR has been shown to be a reliable tool for use during cardiovascular exercise because of its close relation with oxygen consumption, the use of HR as a way of estimating the levels of intensity of training during strength exercises or exercises involving intense participation of the upper limbs has been the subject of controversy. It has been shown that HR has a low correlation with VO_2_ during weight training [34,35], as the number of repetitions and the work duration plays a central role in the increase of HR during exercise. In addition, specific exercises that require a high level of contractions in the upper limbs during resistance exercise solicit a greater HR compared with VO_2_ [35], and the presented exercise protocol had at least one upper-body exercise every round. This cannot be discounted in this study, meaning that the high HR response might be a combination of true O_2_ demand by working muscle, as well as additional HR response as a result of breath holds and thoracic pressure changes, causing changes in the sinus rhythm and HR response. Yet, these results also point to the value of FFT as being more advantageous than traditional intermittent resistance exercises for improvements in aerobic conditioning, and that a hard RPE intensity can produce similar HR responses as an all-out intensity approach.

The use of RPE as a method to control the training intensity could provide an alternative for participants and coaches to reduce the training intensity, and thus provide a training stimulus from which recovery will not be impaired. As the RPE scale in this study was a combination/modification of previously established scales [12,18,19], caution must be taken when generalizing these results. Particularly, it is possible that the inclusion of color and illustrations in the scale utilized in this study can be associated with the participants’ responses, and a greater variability in responses might occur with other versions of the scale. Nevertheless, as the use of the scale presented in this study is an inexpensive, non-invasive method of self-monitoring training intensity during metabolic conditioning sessions of FFT that correlates with LAC and with the number of repetitions completed, practitioners are encouraged to adopt it.

## 5. Conclusions

This study demonstrates that RPE may be a useful tool to prescribe and control training intensity during the metabolic conditioning sessions of functional fitness as a result of its large correlation with lactate and the number of repetitions completed. These findings are of importance in a practical setting, suggesting that coaches could use this method to prescribe the training intensity in a practical and inexpensive way. This allows coaches and practitioners to better manipulate the training loads, and therefore obtain better results, while avoiding negative outcomes, such as excessive fatigue and non-functional overreaching.

## Figures and Tables

**Table 1:** Mean ± standard deviation (SD) of number of repetitions for the all-out and rating of perceived exertion of six (RPE) sessions.

	All-Out	RPE6	Δ (%)	*p*-Value	ES
Round 1	63.9 ± 4.4	46.6 ± 5.8 *	27.1%	≤0.0005 *	3.36
Round 2	58.0 ± 7.7	46.4 ± 7.0 *	20%	0.006 *	1.58
Round 3	41.9 ± 6.6	48.0 ± 1.9 *	−14.5%	0.049 *	1.26
Round 4	50.6 ± 6.5	49.5 ± 4.0	2.2%	0.663	0.20
Total	214.4 ± 18.6	190.5 ± 12.5 *	11.1%	0.020 *	1.51

ES—effect size. * *p* ≤ 0.05 for the all-out session.
